# Limosilactobacillus reuteri SLZX19-12 Protects the Colon from Infection by Enhancing Stability of the Gut Microbiota and Barrier Integrity and Reducing Inflammation

**DOI:** 10.1128/spectrum.02124-21

**Published:** 2022-06-06

**Authors:** Jianmin Wu, Zishen Lin, Xian Wang, Ying Zhao, Jinbiao Zhao, Hu Liu, Lee J. Johnston, Lin Lu, Xi Ma

**Affiliations:** a State Key Laboratory of Animal Nutrition, College of Animal Science and Technology, China Agricultural University, Beijing, China; b College of Animal Science and Technology, Beijing University of Agriculture, Beijing, China; c West Central Research & Outreach Center, University of Minnesota, Morris, Minnesota, USA; National Health Research Institutes

**Keywords:** Tibetan piglets, *Limosilactobacillus reuteri*, colon, infection, *Salmonella* Typhimurium SL1344, host defense, gut microbiota, *Lactobacillus reuteri*

## Abstract

Limosilactobacillus reuteri plays an important role in regulating intestinal functions and maintaining barrier integrity in animals. In this study, Limosilactobacillus reuteri strain SLZX19-12 was isolated from the fecal microbiota of Tibetan pigs, and it was found that this strain is sensitive to common antibiotics and has strong resistance to stress. Upon being administered by gavage at different doses, including low, medium, and high doses, for 14 days, Limosilactobacillus reuteri SLZX19-12 may enhance the intestinal barrier. After administration of a high dose of SLZX19-12, mice were challenged with Salmonella enterica serovar Typhimurium SL1344. Infection with Salmonella Typhimurium SL1344 led to disordered colonic microbiotas, colonic inflammation through the S100A8/S100A9–NF-κB pathway and potential apoptosis, and translocation of pathogens to parenteral visceral organs in mice. However, the mice pretreated with Limosilactobacillus reuteri SLZX19-12 showed lower loads of Salmonella in visceral organs, less colonic inflammation, and higher barrier integrity. More importantly, the administration of strain SLZX19-12 resulted in a more stable microbiota structure of the colon, in which the abundance of *Alloprevotella* was greatly enhanced. Therefore, this study suggests that Limosilactobacillus reuteri SLZX19-12 can protect the colon from infection by enhancing the stability of gut microbiota and barrier integrity and reducing inflammation.

**IMPORTANCE** The use of antibiotics to treat bacterial infections leads to a series of side effects. As an alternative method, the biocontrol strategy, which uses probiotics to suppress pathogens, is considered a potential way to deal with bacterial infections in gut. However, there are few probiotics that are currently safe and can protect against infection. In this study, Limosilactobacillus reuteri strain SLZX19-12 was obtained from Tibetan pigs, which have higher resistance to infection. This strain is sensitive to conventional antibiotics, secretes a wide spectrum of enzymes, and also promotes the intestinal barrier function in mice. In addition, Limosilactobacillus reuteri SLZX19-12 can promote the stability of the gut microbiota to avoid or alleviate the occurrence or development of foodborne infections.

## INTRODUCTION

Antibiotics have played an important role in preventing and treating bacterial infections in humans and farm animals. Long-term use of antibiotics has caused gastrointestinal microorganism disorders, emergence of drug resistance in bacteria, and environmental pollution. These outcomes have increased the occurrence of infections with antibiotic-resistant bacteria, diarrhea, and enteritis (1–3). These negative outcomes have led researchers to search for safe and reliable alternatives to antibiotics. At present, a biocontrol strategy that uses probiotics to suppress pathogens is considered a viable approach to deal with bacterial infections in the gut (4).

The gut is the first protective barrier for animals. Altered permeability of the intestinal barrier can lead to introduction of multiple antigens into the systemic circulation, thereby inducing systemic inflammation and damage to parenteral organs (5). Damage of the intestinal barrier is an important contributor to celiac disease, inflammatory bowel disease, and irritable bowel syndrome (6). Furthermore, development of many autoimmune diseases, such as diabetes, high blood pressure, and arthritis, is related to compromised gut barrier function (7). Therefore, protecting the intestinal mucosa from injury is a key step in the process of disease prevention and control. Intestinal commensal probiotics play an important role in maintaining the intestinal health of the host (8, 9). Colonization by a sufficient number of probiotics in the gut inhibits the subsistence and proliferation of pathogenic bacteria, which prevents disruption of the intestinal mucosal barrier function (10, 11). A gut microbiota that contains a critical number of pathogenic organisms is detrimental to the host. A gut microbiota disturbed by pathogenic organisms first exhibits impaired function and then damages homeostasis of distal organs and tissues through the gut-target organ axes (12).

Some common foodborne bacteria with strong pathogenicity, such as Salmonella enterica serovar Typhimurium, can cause acute infection and sepsis, resulting in animal death. In humans, pigs, mice, and other mammals, *Lactobacillus* can secrete antimicrobial substances and regulate immune defense functions and the gut microbiota to prevent infectious diseases (13–16). Limosilactobacillus reuteri, formerly known as Lactobacillus reuteri, is a lactobacillus with excellent performance, which can inhibit inflammation, strengthen intestinal barrier function, maintain intestinal regeneration, and repair damaged intestinal mucosa (17, 18). L. reuteri has multiple ways to kill pathogens, including secreting antibacterial substances such as organic acids, ethanol, and reuterin (19). However, there is a lack of further research on the other mechanisms of L. reuteri in regulating the gut microbiota. Even within the same species, different strains of L. reuteri show diverse biological traits (20, 21). In the digestive tract of animals with strong resistance to infection, there may be some probiotics with special functions to promote tolerance to pathogens (22). Therefore, obtaining probiotic resources from the digestive tracts of animals with strong resistance to infection and using them to clarify the mechanism of disease resistance and explore their application in preventing intestinal diseases constitute a promising but challenging field.

In a recent study (our unpublished data), we discovered a high abundance of L. reuteri in Tibetan pigs with high resistance to a hostile environment, including cold temperatures, low oxygen levels, and high concentrations of UV rays. Tibetan pigs can survive in the wild by eating weeds, and they are prone to contact with pathogenic microorganisms. However, infectious diseases are rarely acquired in Tibetan pigs in this environment. Considering the disease resistance and living habits of the Tibetan pigs, we hypothesized that this strain of L. reuteri could strengthen the intestinal barrier and maintain intestinal health. L. reuteri strain SLZX19-12 was isolated from the feces of Tibetan piglets. An oral trial indicated that L. reuteri SLZX19-12 improves the intestinal barrier function in mice. An infection trial showed that L. reuteri SLZX19-12 has superior ability to prevent the invasion by *S.* Typhimurium SL1344 of the colon and can defend against the inflammation induced by the S100A8/S100A9–NF-κB pathway and potential apoptosis, in which the enhanced stability of the gut microbiota may be one of the main mechanisms.

## RESULTS

### L. reuteri SLZX19-12 improved gene expression levels of colonic barrier proteins.

**FIG 1:**
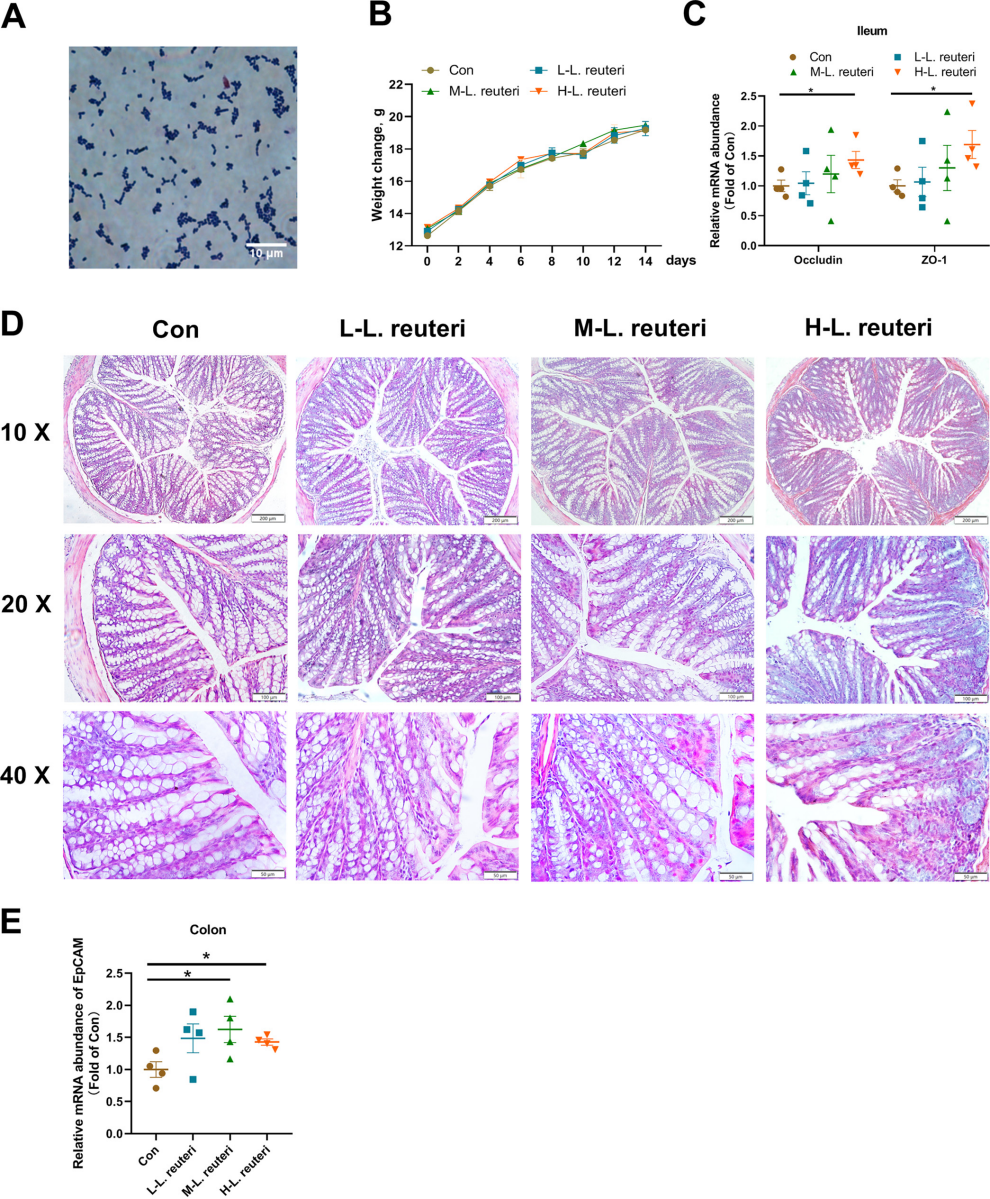
Regulation by L. reuteri SLZX19-12 of the intestinal barrier of mice. (A) Morphological characteristics of L. reuteri SLZX19-12 visualized by Gram staining. (B) Body weights of mice during gavage with L. reuteri SLZX19-12. Mice were treated with vehicle (Con group) or a low dose (L-L. reuteri group), medium dose (M-L. reuteri group), or high dose (H-L. reuteri group) of L. reuteri SLZX19-12. On the *x* axis, “days” refers to the time during the trial. The mice were weighed every 2 days until day 14. Means and SE are shown (*n* = 4). (C) Relative gene expression levels of the barrier proteins occludin and ZO-1 determined by RT-qPCR. Means and SE are shown (*n* = 4) (*, *P* < 0.05). (D) HE staining of colon tissue sections of mice to view the general morphological changes. (E) Relative expression levels of the EpCAM gene, determined by RT-qPCR. Means and SE are shown (*n* = 4) (*, *P* < 0.05).

Based on enrichment culture and screening of bacteria producing acids, we successfully isolated the desired strain, L. reuteri SLZX19-12 (Fig. 1A). This strain had superior growth (see Fig. S1A and B in the supplemental material). In the plateau stage, the number of bacteria reached 1 × 10^8^ CFU/mL (Fig. S1B). L. reuteri SLZX19-12 was sensitive to common antibiotics, including ampicillin (β-lactam antibiotic), neomycin (aminoglycoside antibiotic), and minocycline (tetracycline antibiotic) (Fig. S1C). In the tolerance test, L. reuteri SLZX19-12 showed about 50% survival in a pH 2 environment for 1 h (Fig. S1D) and a >10% survival rate in 0.1 g/100 mL bile salt for 4 h (Fig. S1E). According to the biochemical identification test, L. reuteri SLZX19-12 was found to hydrolyze cellobiose, maltose, sucrose, raffinose, and lactose but not aescinate, mannitol, salicin, sorbitol, inulin, and sodium hippurate (Fig. S1F). L. reuteri SLZX19-12 can produce a wide spectrum of enzymes, including esterase C4, lipase-like C8, albumin arylaminase, acid phospholipase, naphthol AS-BI-phosphate hydrolase, α-galactosidase, β-galactosidase, and α-glucosidase (Fig. S1G).

After gavage with L. reuteri SLZX19-12 for 14 days, there was no significant difference in body weight of mice (Fig. 1B). In the ileum, gene expression levels of the barrier proteins occludin and ZO-1 were increased compared to the control (Con) group (Fig. 1C). The changes in colon tissue from mice receiving low, medium, and high doses of L. reuteri SLZX19-12 (L-L. reuteri, M-L. reuteri, and H-L. reuteri groups, respectively) are shown in Fig. 1D. All groups of mice showed no significant changes in the gene expression levels of tumor necrosis factor alpha (TNF-α) and interleukin-1β (IL-1β) and IL-10 in colon (Fig. S2A). Epithelial cell adhesion molecule (EpCAM) plays a positive role in tight junctions of the gut (23). In the M-L. reuteri and H-L. reuteri groups, the gene expression level of EpCAM in the colon significantly increased compared to that in the Con group (Fig. 1E). The gene expression levels of the barrier proteins occludin and ZO-1 in the colon were not significantly changed compared to those in the Con group (Fig. S2B).

### L. reuteri SLZX19-12 reduced the signs of infection.

**FIG 2:**
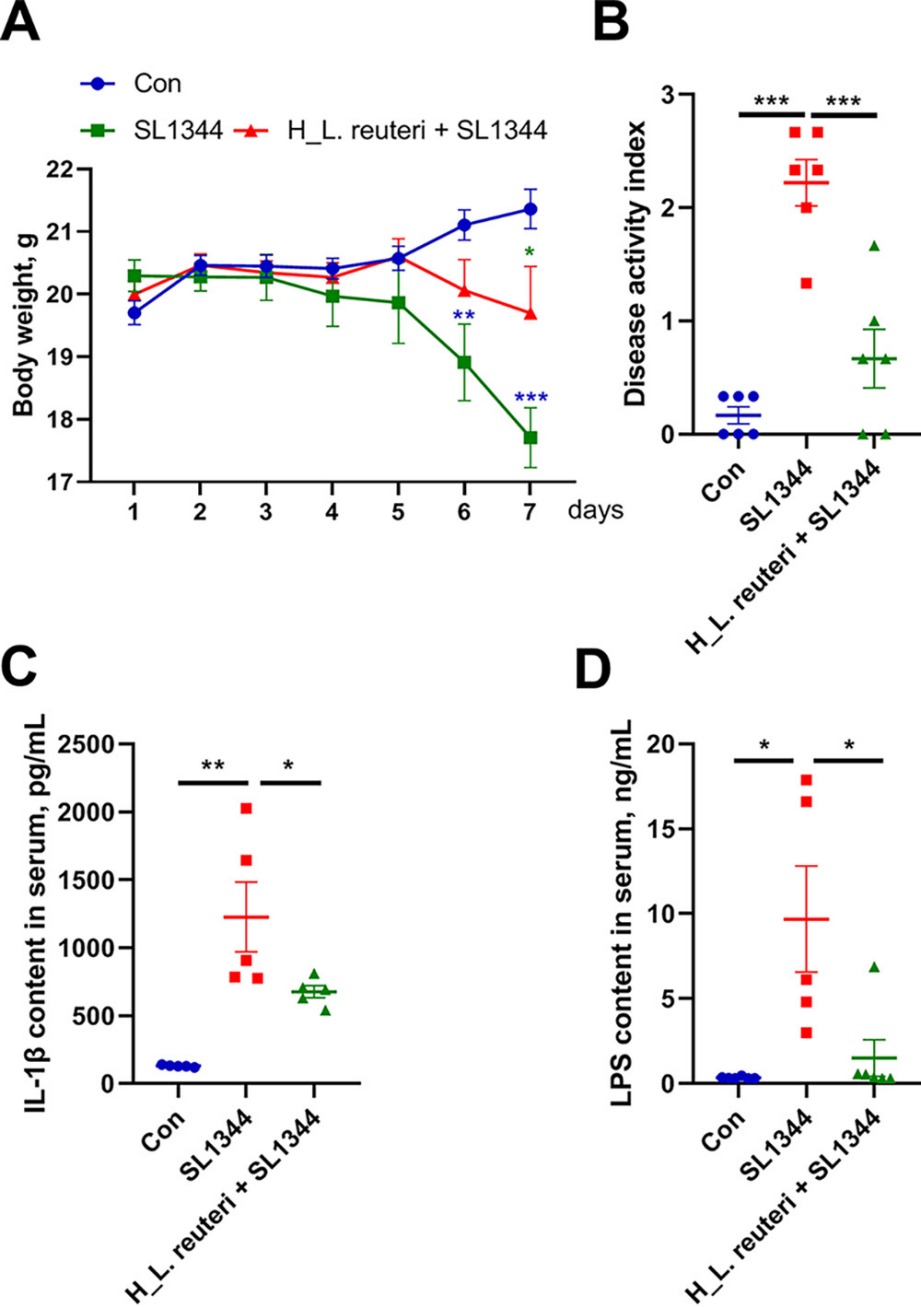
Preventive effects of L. reuteri SLZX19-12 on colons of mice infected with *S.* Typhimurium SL1344. (A) Body weights after the challenge with *S.* Typhimurium SL1344. Mice were treated with vehicle (Con and SL1344 groups) or a high dose of L. reuteri SLZX19-12 (H_L. reuteri+SL1344 group) and then given vehicle (Con group) or infected with *S.* Typhimurium SL1344 (SL1344 and H_L. reuteri+SL1344 groups). On the *x* axis, “days” refers to the time after challenge with *S.* Typhimurium SL1344. After infection, the mice were weighed daily until day 7. Means and SE are shown (*n* = 6) (*, *P* < 0.05; **, *P* < 0.01; ***, *P* < 0.01). (B) DAIs at day 7 after challenge with *S.* Typhimurium SL1344. DAIs were determined according to the scoring standard in the case of unknown experimental treatment. A high DAI score indicates more severe disease symptoms. Means and SE are shown (*n* = 6) (***, *P* < 0.01). (C) Proinflammatory factor IL-1β content in serum, determined by ELISA. Higher levels of proinflammatory factors reflect stronger inflammation in the body. Means and SE are shown (*n* = 6) (*, *P* < 0.05; **, *P* < 0.01). (D) LPS content of serum, determined by ELISA. Disruption of intestinal barrier function could result in enterically derived LPS entering the bloodstream. Means and SE are shown (*n* = 6) (*, *P* < 0.05).

To determine the effect of L. reuteri SLZX19-12 on foodborne pathogenic bacteria, we chose strongly pathogenic *S.* Typhimurium SL1344 to infect mice. On day 7 after infection, body weights of mice in the SL1344 group decreased significantly compared to those in the Con group (Fig. 2A). However, compared with SL1344 group, the infected mice pretreated with a high dose of L. reuteri SLZX19-12 (H_L. reuteri+SL1344) showed less weight loss (Fig. 2A). On days 6 and 7 after infection, body weight in the H_L. reuteri+SL1344 group was significantly higher than that in the SL1344 group (Fig. 2A). The SL1344 group had a higher disease activity index (DAI) than the Con group (Fig. 2B). The DAI was lower in the H_L. reuteri+SL1344 group than in the SL1344 group (Fig. 2B). Consistent with the DAI score, the IL-1β content of serum of the SL1344 group was significantly increased compared to that in the Con group (Fig. 2C). Similarly, the H_L. reuteri+SL1344 group showed a lower level of IL-1β content in serum than the SL1344 group (Fig. 2C). In serum, the lipopolysaccharide (LPS) content was significantly higher in the SL1344 group than in the Con and H_L. reuteri+SL1344 groups (Fig. 2D).

### L. reuteri SLZX19-12 decreased Salmonella load in gut and restricted its translocation.

**FIG 3:**
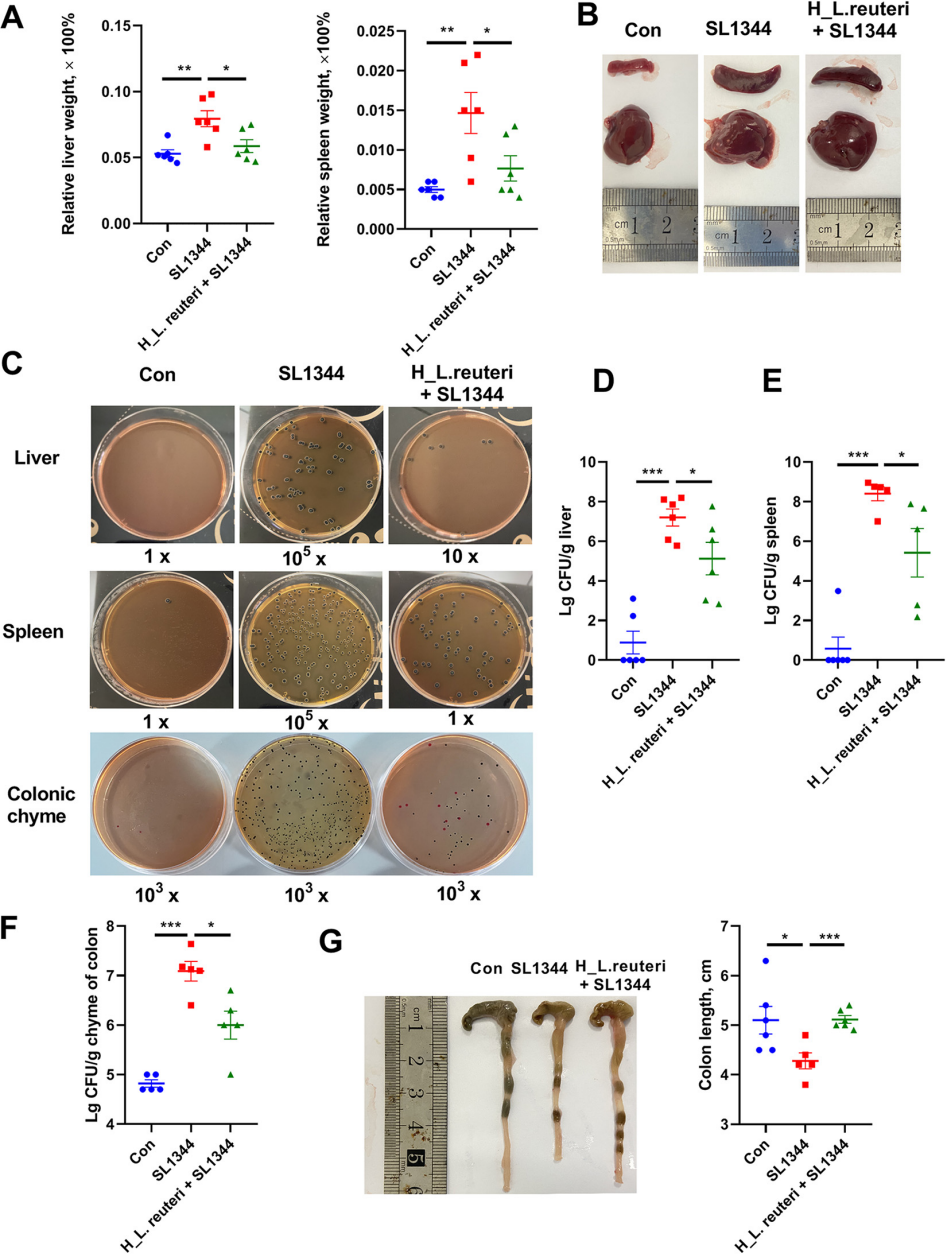
Effects of L. reuteri SLZX19-12 on colonic barrier function of mice infected with *S.* Typhimurium SL1344. (A and B) Relative weights of livers (A) and spleens (B). These indexes were calculated by tissue weight/body weight (both in grams). The relative organ weights indicate the degree of swelling of the liver and spleen, which can reflect an enhanced immune response. Means and SE are shown (*n* = 6) (*, *P* < 0.05; **, *P* < 0.01). (C) Loads of *S.* Typhimurium in liver, spleen, and colonic chyme. Colonic chyme and tissue were homogenized and serially diluted to 1×, 10×, 100×, 10^3^×, 10^4^×, and 10^5^×. After spreading of samples on SS agar plates and growth, viable colony counts ranged from 30 to 300 per plates. The black colonies are Salmonella, and the red ones are E. coli. (D to F) Loads of Salmonella in liver (D), spleen (E), and colonic chyme (F). Means and SE are shown (*n* = 6) (*, *P* < 0.05; ***, *P* < 0.01). (G) Colon length of mice, measured using a ruler. Means and SE are shown (*n* = 6) (*, *P* < 0.05; ***, *P* < 0.01).

To further evaluate the integrity of the gut, bacterial translocation was detected. According to the relative organ weight and morphology images, the SL1344 group showed significantly higher liver and spleen indexes than the Con group (Fig. 3A and B). However, compared with SL1344 group, the H_L. reuteri+SL1344 group had appreciably reduced liver and spleen indexes (Fig. 3A and B). The representative images in Fig. 3B show changes in morphology of livers and spleens. Meanwhile, the pathogens loads in the liver were assessed with Salmonella-*Shigella* (SS) agar medium. Similar to the morphology, loads of Salmonella in the livers (Fig. 3C and D) and spleens (Fig. 3C and E) of the SL1344 group were significantly increased compared to those in the Con group. Mice in the H_L. reuteri+SL1344 group exhibited lower loads of Salmonella in liver and spleen than those in the SL1344 group (Fig. 3C to E). After infection, the load of Salmonella in the colonic chyme of the SL1344 group was higher than that in the Con group (Fig. 3C and F), and the colons of the SL1344 group were shorter than those of the Con group (Fig. 3G). However, the H_L. reuteri+SL1344 group exhibited a longer colon and lower load of Salmonella in colonic chyme than the SL1344 group (Fig. 3C, F, and G).

### L. reuteri SLZX19-12 inhibited infection-induced colitis.

**FIG 4:**
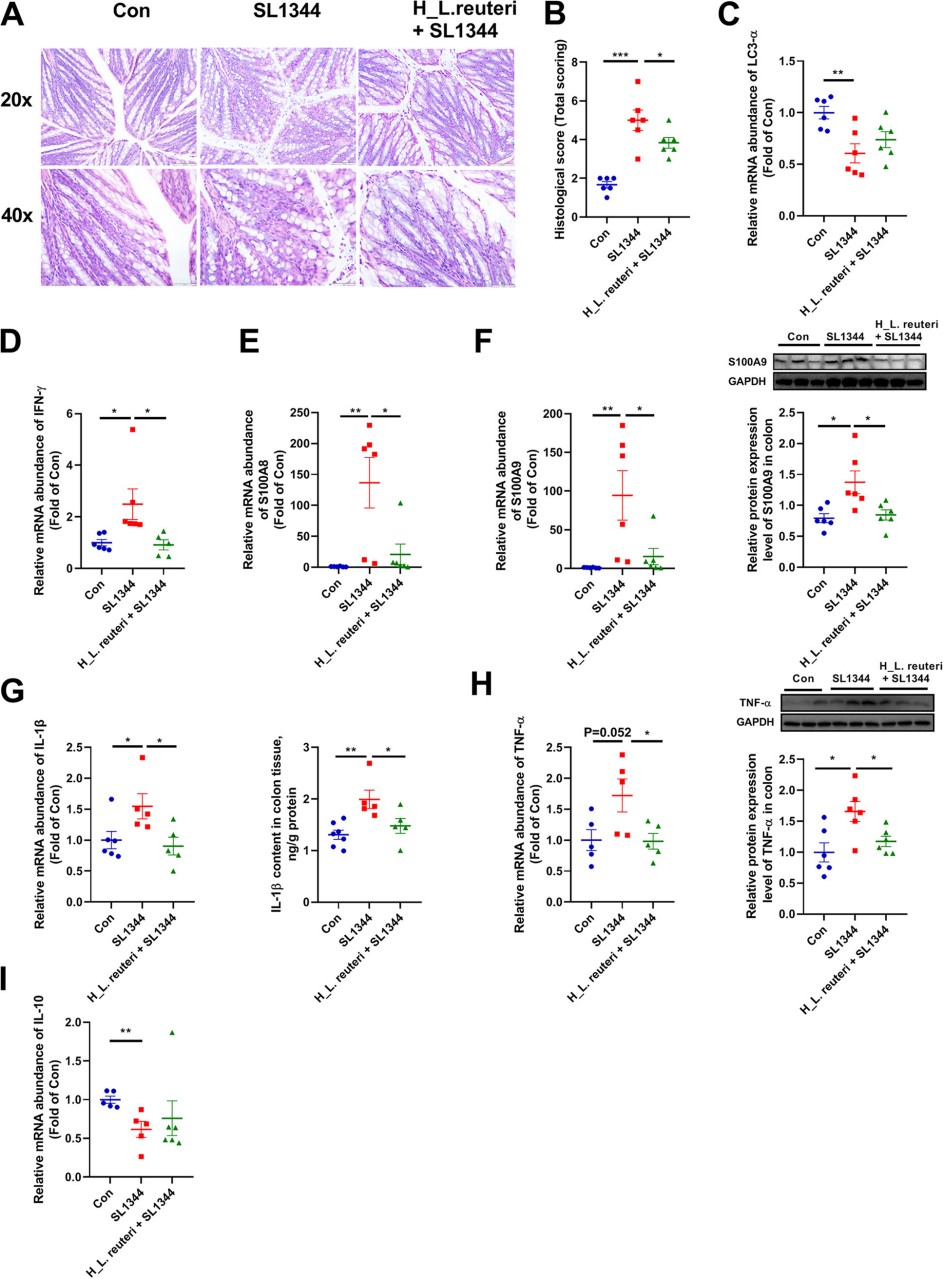
Effects of L. reuteri SLZX19-1*2* on colonic morphology, autophagy function, inflammatory factors, and barrier proteins of mice infected with *S.* Typhimurium SL1344. (A) Morphological characteristics of colon tissue visualized by HE staining. (B) Histology was scored according to the scoring standard in the case of unknown experimental treatment. A high histology score indicates more severe colon lesions. Means and SE are shown (*n* = 6) (*, *P* < 0.05; ***, *P* < 0.01). (C to E and I) Relative expression levels of LC-3α (C), IFN-γ (D), S100A8 (E), and IL-10 (I) genes by RT-qPCR. Means and SE are shown (*n* = 6) (*, *P* < 0.05; **, *P* < 0.01). (F to H) Relative gene and protein expression levels of S100A9, IL-1β, and TNF-α. The relative gene expression levels of S100A9 (F), IL-1β (G), and TNF-α (H) were detected by RT-qPCR. The normalized relative protein expression levels of S100A9 (F) and TNF-α (H) were measured by Western blotting. The protein expression level of IL-1β (F) was checked with an ELISA kit. Means and SE are shown (*n* = 6) (*, *P* < 0.05; **, *P* < 0.01).

We acquired tissue from the fixed middle position of colons for sections and hematoxylin-and-eosin (HE) staining. From the representative images, the colon interstitium of the SL1344 group exhibited irregular and damaged epithelium, larger and fewer goblet cells, and more inflammatory cell infiltration than that of the Con group (Fig. 4A). Compared with the SL1344 group, the H_L. reuteri+SL1344 group possessed more intact epithelial structure and more crypts and showed more goblet cells and fewer inflammatory cell infiltrates (Fig. 4A). Histological scoring was performed to quantify these changes in colonic pathology symptoms. Compared to the Con group, the mice in the SL1344 group showed significantly increased histological scores (Fig. 4B). However, the H_L. reuteri+SL1344 group exhibited appreciably reduced scores compared to the SL1344 group (Fig. 4B).

To clarify the mechanism by which L. reuteri SLZX19-12 prevents the invasion of the colon by *S.* Typhimurium SL1344, we detected expression levels of key genes or proteins involved in autophagy, apoptosis, and the inflammatory process. Light chain 3 (LC3) is a marker of autophagy whose function is mainly involved in the formation of autophagosomes (24). Relative expression level of the *LC3-α* gene in the SL1344 group was lower than that in the Con group (Fig. 4C). However, there was no significant difference between the H_L. reuteri+SL1344 and SL1344 groups in *LC3-α* expression (Fig. 4C).

Innate immunity is the body's first line of defense against pathogens. By checking the innate immune pathways, we found that the expression level of the gamma interferon (IFN-γ) gene was significantly increased in the SL1344 group compared to the Con group (Fig. 4D). However, the H_L. reuteri+SL1344 group exhibited a lower mRNA level of *IFN-γ* (Fig. 4D). Transforming growth factor β (TGF-β), regenerating islet-derived protein IIIγ (REGIIIγ), IL-12, IL-18, IL-22, IL-23, CXCL1, and CXCL2 also play important roles in immune regulation of the mucosa (25–27). However, the mRNA levels of these genes were not statistically different among groups in this study (Fig. S2C). Calcium-binding proteins S100A8 and S100A9, which belong to the S100 protein family and are involved in anti-infective and proinflammatory responses in the form of S100A8/S100A9 heterodimers, are viewed as the alarmins and biomarkers of inflammatory bowel disease (28). In the SL1344 group, the mRNA and protein levels of S100A8 and S100A9 were strongly upregulated compared with the other two groups (Fig. 4E and F). The IL-1β gene expression level was clearly higher in the SL1344 group than the Con group (Fig. 4G). Meanwhile, mRNA and protein levels of TNF-α in the SL1344 group showed similarly increased trends or differences (Fig. 4H). However, the IL-10 gene expression level was clearly lower in the SL1344 group than the Con group (Fig. 4I). In contrast, the gene and protein expression levels of TNF-α and IL-1β of the H_L. reuteri+SL1344 group were lower than those in the SL1344 group (Fig. 4G and H).

**FIG 5:**
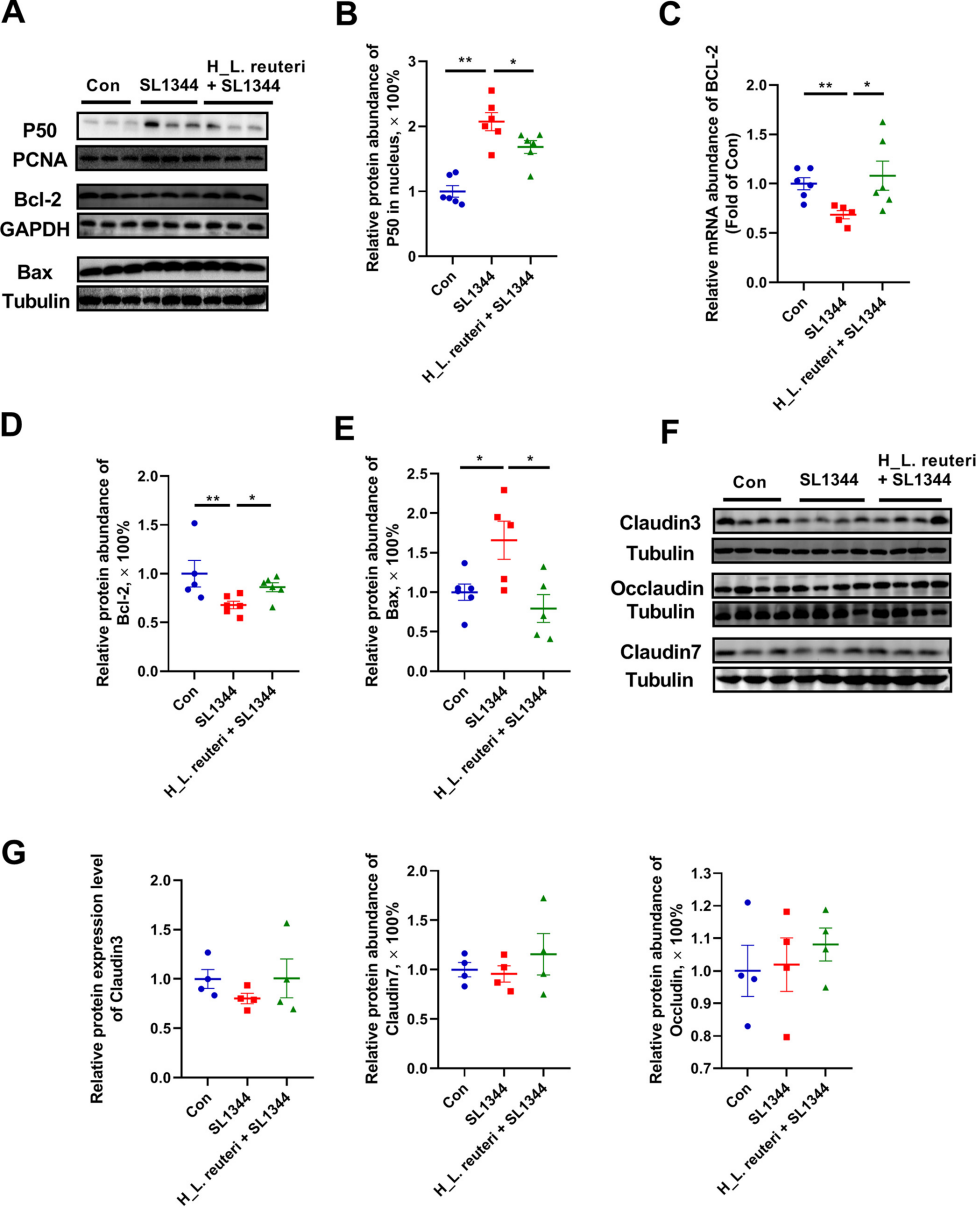
Effects of L. reuteri SLZX19-12 on NF-κB and apoptosis pathways and expression levels of barrier proteins in mice infected with *S.* Typhimurium SL1344. (A) Bands of endonuclear NF-κB P50 and total Bcl-2 and Bax protein in the colon. The endonuclear proteins were extracted with a commercial nuclear and cytoplasmic protein extraction kit. All endonuclear protein samples and total protein samples were detected by Western blotting. (B) Relative protein expression levels of endonuclear NF-κB P50. Means and SE are shown (*n* = 6) (*, *P* < 0.05; **, *P* < 0.01). (C) Relative expression levels of the Bcl-2 gene determined by RT-qPCR. Means and SE are shown (*n* = 6) (*, *P* < 0.05; **, *P* < 0.01). (D and E) Normalized relative protein expression levels of Bcl-2 (D) and Bax (E). Means and SE are shown (*n* = 6) (*, *P* < 0.05; **, *P* < 0.01). (F) Western blot showing bands of claudin-3, claudin-7, and occludin protein from the colon. (G) Normalized relative protein expression levels of claudin-3, claudin-7, and occludin. Means and SE are shown (*n* = 4).

In the study of the disease resistance mechanism, the level of colonic NF-κB p50 of the SL1344 group was significantly higher in the cell nucleus than that in the Con or H_L. reuteri+SL1344 group (Fig. 5A and B). The infection and inflammation environment can lead to apoptosis of enterocytes and destruction of the epithelial barrier, in which Bcl-2-associated X (Bax) protein and B-cell lymphoma-2 (Bcl-2) protein, respectively, contribute to and inhibit the development of apoptosis (29, 30). The SL1344 group showed significantly lower gene and protein expression levels of Bcl-2 than the Con group (Fig. 5A, C, and D). However, gene and protein expression levels of Bcl-2 were higher in the H_L. reuteri+SL1344 group than the SL1344 group (Fig. 5A, C, and D). In contrast, protein expression levels of Bax decreased in the SL1344 group compared to the Con group. Relative to the SL1344 group, the protein expression level of Bax in the H_L. reuteri+SL1344 group was significantly recovered (Fig. 5A and E). In the measurement of proteins participating in formation of epithelial barriers, expression levels of occludin, claudin-3, claudin-7, and EpCAM did not change in the SL1344 group compared to the Con and H_L. reuteri+SL1344 groups (Fig. 5F and G and Fig. S2C).

### L. reuteri SLZX19-12 strengthened the stability of gut microbiota.

**FIG 6:**
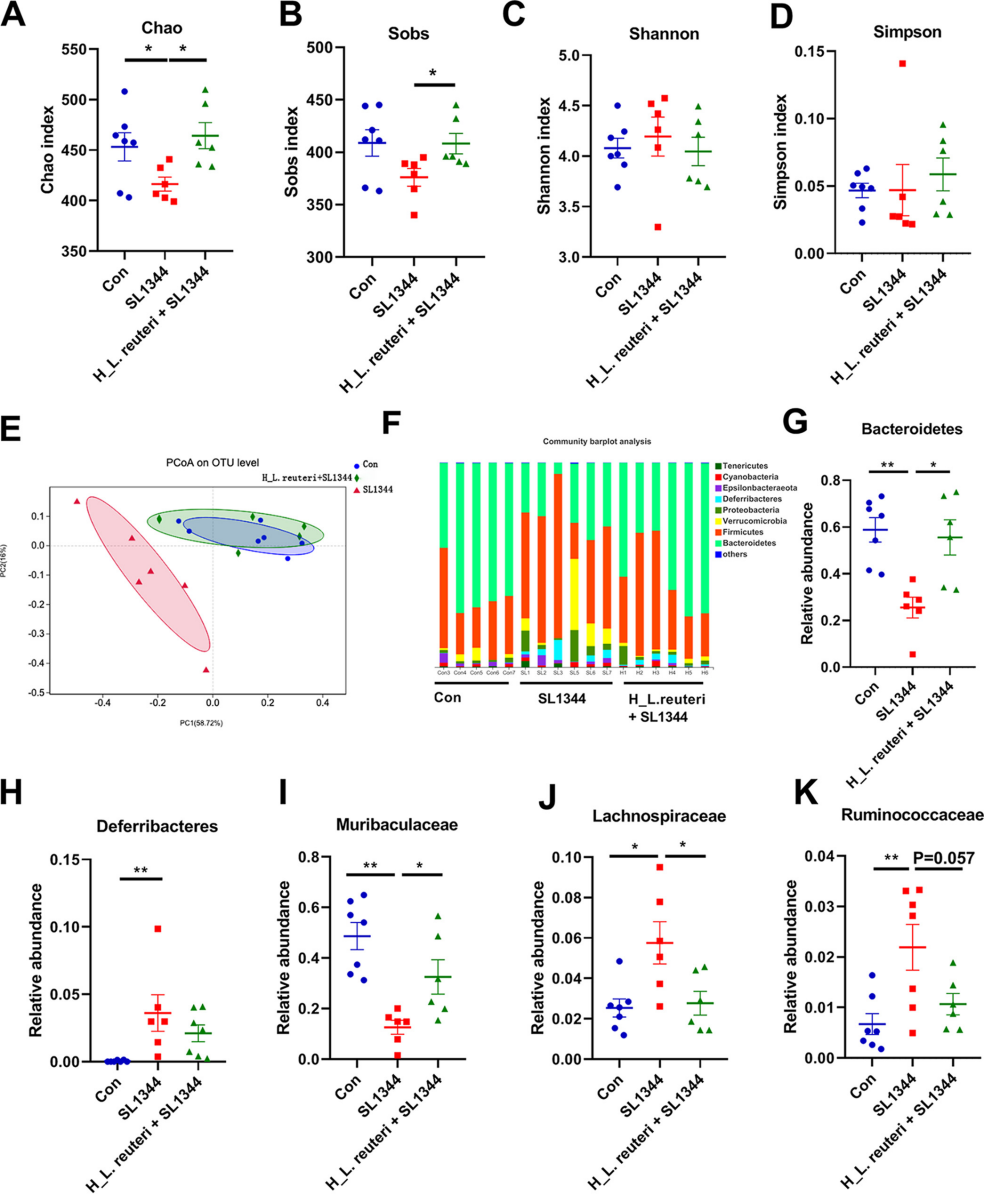
Effects of L. reuteri SLZX19-12 on diversity and community abundance at the phylum level in the colonic microbiota of mice infected by *S.* Typhimurium SL1344. (A to D) Chao (A), Sobs (B), Simpson (C), and Shannon (D) indexes of the colonic microbiota. These indexes evaluate α diversity. Means and SE are shown (*n* = 7) (*, *P* < 0.05). (E) PCoA of the colonic microbiota based on the weighted normalized UniFrac method in mice. PCoA evaluates β diversity. (F) Community abundances of colonic microbiota at the phylum level in mice. The eight most abundant phyla are shown. (G to K) Community abundances of *Bacteroidetes* (G), *Deferribacteres* (H), *Muribaculaceae* (I), *Lachnospiraceae* (J), and *Ruminococcaceae* (K) of the colonic microbiota in mice. Means and SE are shown (*n* = 7) (*, *P* < 0.05; **, *P* < 0.01).

In view of the results above, we suspected that the gut microbiota might play a key role in preventing infection by *S.* Typhimurium SL1344. Microorganism composition and structure in the colons of mice were analyzed comprehensively by assessing 16S rRNA genes. The α diversity of colonic microbiota was elevated by Sobs, Chao, Shannon, and Simpson indexes. Compared with the Con group, Shannon and Simpson indexes in the SL1344 group did not change, but the Chao index decreased (Fig. 6A to D). However, compared to the SL1344 group, the H_L. reuteri+SL1344 group showed significantly increased Sobs and Chao indexes (Fig. 6A and B). According to β diversity, principal-coordinate analysis (PCoA) based on the weighted normalized UniFrac method showed that the colonic community composition of the SL1344 group was significantly distinct from that of the Con and H_L. reuteri+SL1344 groups (Fig. 6E). However, the H_L. reuteri+SL1344 and Con groups exhibited similar colonic community composition features (Fig. 6E).

**FIG 7:**
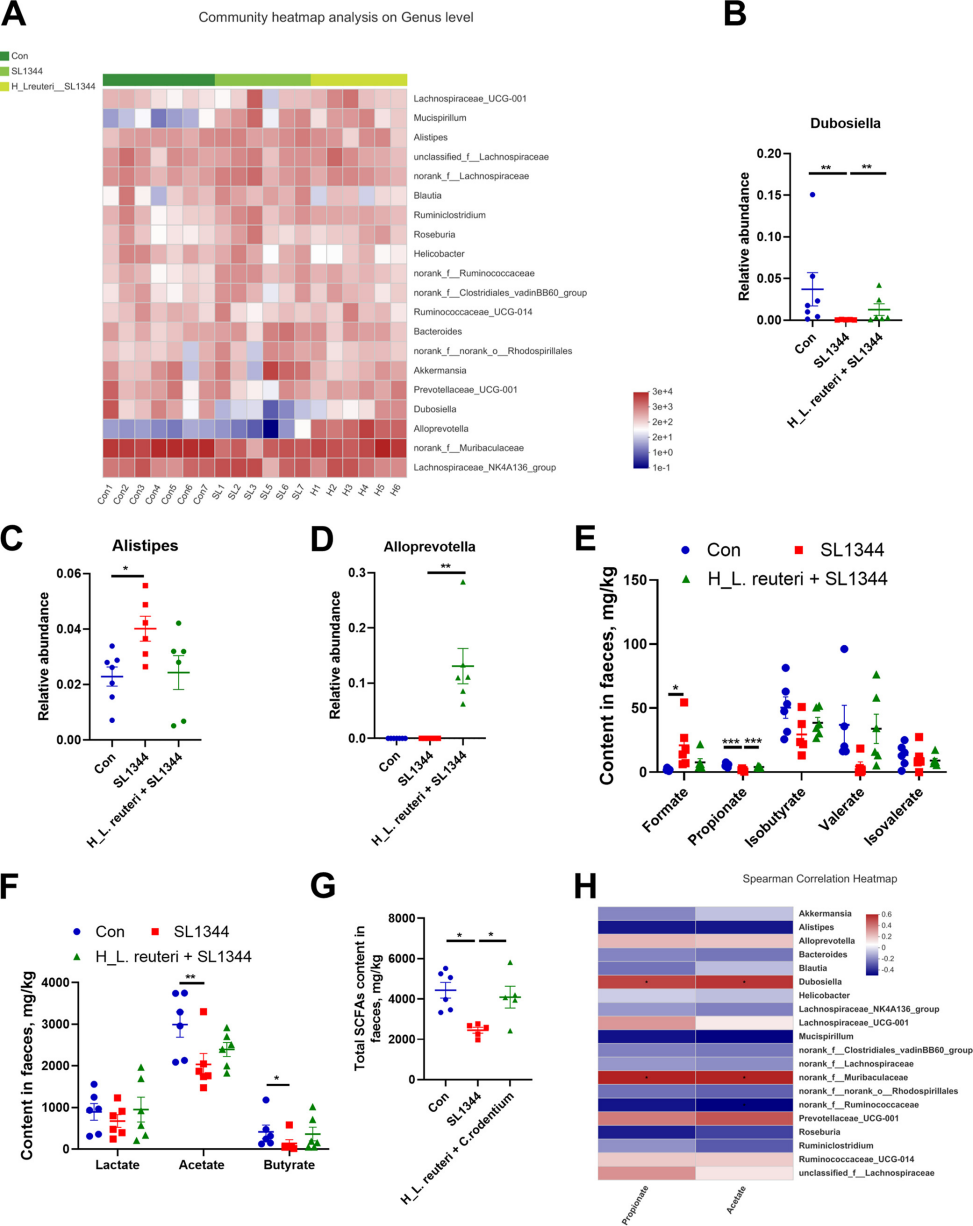
Effects of L. reuteri SLZX19-12 on community abundance at the genus level in the colonic microbiota of mice infected with *S.* Typhimurium SL1344. (A) Community heat map analysis of colonic microbiota at the genus level in mice. This heat map shows the 20 most abundant communities. (B to D) Community abundances of *Dubosiella* (B), *Alistipes* (C), and *Alloprevotella* (D) in the colonic microbiota of mice. Means and SE are shown (*n* = 7) (*, *P* < 0.05; **, *P* < 0.01). (E) Formate, propionate, isobutyrate, valerate, and isovalerate content in feces of mice. SCFAs were detected by ion chromatography. Means and SE are shown (*n* = 6) (*, *P* < 0.05; ***, *P* < 0.001). (F) Lactate, acetate, and butyrate content in feces. Means and SE are shown (*n* = 6) (*, *P* < 0.05; **, *P* < 0.01). (G) Total SCFA content in feces. The total SCFA content was calculated by adding the formate, propionate, isobutyrate, valerate, isovalerate, lactate, acetate, and butyrate values together. Means and SE are shown (*n* = 6) (*, *P* < 0.05). (H) Heat map of correlation analysis between fecal acetate and propionate and the colonic microbiota. The analysis method was based on Spearman’s coefficient of rank correlation (*n* = 7) (*, *P* < 0.05).

At the phylum level, infection of *S.* Typhimurium SL1344 significantly decreased colonic *Bacteroidetes* and increased *Deferribacteres* abundances in the SL1344 group relative to the Con group (Fig. 6F and G). In contrast, compared to the SL1344 group, the H_L. reuteri+SL1344 group had appreciably higher abundance of *Bacteroidetes* (Fig. 6F and G). *Deferribacteres* abundance was reduced in the H_L. reuteri+SL1344 group compared to the SL1344 group (Fig. 6F and H). At the family level, the SL1344 group exhibited remarkably decreased colonic *Muribaculaceae* abundance but higher *Lachnospiraceae* and *Ruminococcaceae* abundances relative to the other two groups (Fig. 6I to K). Compared with the SL1344 group, the H_L. reuteri+SL1344 group had significantly higher *Muribaculaceae* abundance and showed a trend of reduced *Ruminococcaceae* abundance (Fig. 6I and K). At the genus level, infection with *S.* Typhimurium SL1344 in the SL1344 group markedly decreased the abundance of colonic *Dubosiella* (Fig. 7A and B) and enhanced the abundance of *Alistipes* compared with the Con group (Fig. 7C). In contrast, pretreatment with L. reuteri SLZX19-12 in the H_L. reuteri+SL1344 group significantly increased the abundant of *Dubosiella* compared to the SL1344 group (Fig. 7B). Of particular concern, considerable abundance of colonic *Alloprevotella* existed in the H_L. reuteri+SL1344 group (Fig. 7D). The abundance of *Alloprevotella* in the H_L. reuteri+SL1344 group was significantly higher than that in the SL1344 group. However, the abundance of *Alloprevotella* was extremely low in the colons of the Con group and SL1344 group (Fig. 7D).

With the changes in abundances of the communities, there were significant changes in the metabolites of colon. In the SL1344 group, the contents of acetate, propionate, and butyrate of feces were significantly decreased, while the content of formate was significantly increased compared to that in the Con group (Fig. 7E and F). In line with this, the total short-chain fatty acid (SCFA) content of the SL1344 group was also reduced compared to that in the Con group (Fig. 7G). The H_L. reuteri+SL1344 group showed a higher content of propionate in feces than the SL1344 group. In addition, the total fecal SCFA content of the H_L. reuteri+SL1344 group was significantly higher than that in the SL1344 group (Fig. 7G). Correlation analysis based on Spearman’s rank correlation coefficient between fecal SCFAs and the colonic microbiota showed that the changes in fecal acetate and propionate contents were significantly correlated with *Dubosiella* and norank_f_*Muribaculaceae* (Fig. 7H).

## DISCUSSION

### L. reuteri SLZX19-12 from Tibetan piglets may enhance intestinal barrier function.

In the background of special environments and during the evolution process, intestinal tissue and the gut microbiota have formed a stable mutualistic relationship and together constitute a symbiont (12). Endosymbiosis can affect stress tolerance of holobionts (31). Based on this view, microorganisms with special functions living in the digestive tracts of animals may exhibit special characteristics. Therefore, the source of probiotics is of great importance. The Tibetan pig is a well-known domestic breed with strong resistance to disease and unique physiological characteristics (32, 33). In the present research, L. reuteri SLZX19-12 was obtained from fecal samples of Tibetan piglets from remote rural and antibiotic-free areas in Tibet and exhibited strong resistance to stress. According to current research progress, drug resistance genes in probiotics can spread through the gut microbiota and pollute the environment (34). L. reuteri SLZX19-12 does not exhibit significant antibiotic resistance and may be reliable in terms of biosafety for widespread use in piglets, humans, and other animals. L. reuteri, commonly found in the intestines of vertebrates, is recognized as a probiotic and has been widely accepted for use in human food and animal feeds (35). Upon gavage with different doses of L. reuteri SLZX19-12, mice presented increased expression levels of *EpCAM*, *Occludin*, and *ZO-1*. In normal physiology, EpCAM localizes to tight junctions, adherens junctions, and the lateral membranes of epithelial cells and regulates adhesive structures between cells and the cell matrix, including tight junctions, adherens junctions, desmosomes, and hemidesmosomes (23, 36). Therefore, increased expression of *EpCAM*, *Occludin*, and *ZO-1* indicates enhanced barrier function. Therefore, it can be inferred that L. reuteri SLZX19-12 from Tibetan piglets may be safe and beneficial for the barrier function of the intestine, including the small intestine and large intestine, under normal conditions.

### L. reuteri SLZX19-12 enhances the anti-infection capacity of the colon.

To prove the protective role of L. reuteri SLZX19-12, mice were challenged with *S.* Typhimurium SL1344. *S.* Typhimurium is a common pathogen in water, food, and living environments. In the intestinal tract, *S.* Typhimurium can cause acute infection, resulting in diarrhea, intestinal inflammation, fecal blood, and sepsis, depending on the serotype or strain involved (37). Digestive tract diseases caused by Salmonella infection in animals and humans are common. In this research, infection with *S.* Typhimurium SL1344 caused rapid weight loss and fecal bleeding in mice, which is similar to previous reports (38). Based on the symptoms, DAI was evaluated and confirmed severe damage in mice in the SL1344 group. Therefore, this dose of *S.* Typhimurium SL1344 successfully infected the mice.

As an internal defense, intestinal tissue clears out Salmonella-infected epithelial cells to reduce the spread of pathogenic bacteria through the intestinal epithelium (39). Thus, the integrity of the epithelial barrier could be destroyed by severe infection with *S.* Typhimurium SL1344, allowing the damaged sites to act as gateways for invasion by pathogenic bacteria. In this research, increased potential apoptosis may have provided similar gateways. An increase in liver and spleen indexes in the SL1344 group indicates that Salmonella had broken the intestinal barrier and caused a strong immune response in the body. According to counts of pathogenic bacteria, numbers of *S.* Typhimurium in the liver and spleen of mice in the SL1344 group were indeed significantly increased, suggesting that these mice were suffering from bacteremia. Similar results were found previously (38, 40).

A significant elevation of serum LPS also verified increased intestinal permeability of mice in the SL1344 group. Relative to the SL1344 group, higher body weight and lower DAI in the H_L. reuteri+SL1344 group suggest that supplementation with L. reuteri SLZX19-12 significantly enhanced the ability of mice to defend against infection. Pretreatment with L. reuteri SLZX19-12 alleviated the immune responses induced by *S.* Typhimurium SL1344. According to the counts of *S.* Typhimurium in tissue, the reduction in liver and spleen indexes may be due to the lower *S.* Typhimurium load. These results indicate that gavage with L. reuteri SLZX19-12 weakened the degree of damage to the intestinal barrier caused by *S.* Typhimurium SL1344 challenge. Compared to the SL1344 group, decreased concentration of LPS in serum of the H_L. reuteri+SL1344 group also confirms the more integrated barrier structures. In accordance with above indexes, mice of the SL1344 group indeed showed a higher histological score in the colon. In contrast, mice of the H_L. reuteri+SL1344 group showed a lower histological score and much less epithelial damage.

Surprisingly, the expression of the barrier proteins occludin, claudin-1, claudin-2, claudin-7, and EpCAM was not significantly influenced by *S.* Typhimurium SL1344, as was reported by Sun et al. (40). According to previous research, Salmonella usually breaks through intestinal epithelial cells to the lamina propria of the mucosa via transcytosis of M cells, epithelial endocytosis, and phagocyte uptake (41–43). In the colon, virulence proteins from Salmonella interact with ordinary intestinal epithelial cells and rearrange the cytoskeleton (44). Skeletal rearrangement may negatively affect formation of intact epithelial barrier structures, resulting in leakage. Based on expression of barrier proteins in our research, we surmise that cytoskeleton rearrangement and invasion of *S.* Typhimurium SL1344 may not influence expression of barrier proteins but may change assembly of barrier structures. Sun et al. (40) indeed found that SpvB in *S.* Typhimurium SL1344 can cause the cellular redistribution of claudin-1, occludin, and E-cadherin junctional proteins. However, how Salmonella interferes with formation of barrier structures remains unclear. Acute Salmonella infection induces production of IFN-γ, which limits recovery from infection (45). By inducing IFN-γ to damage the colon, *S.* Typhimurium SL1344 may cross the gut barrier into the animal body. IFN-γ production in the intestine caused by *S.* Typhimurium SL1344 infection facilitates migration of *S.* Typhimurium in lymph (46).

In recent years, the functions of S100A8 and S100A9 have gained attention (47). S100A8/S100A9 heterodimers are viewed as the diagnostic biomarker and therapeutic target of acute infection and inflammation (47, 48). High levels of expression of *S100A8* and *S100A9* induced by *S.* Typhimurium SL1344 hint that the mice were in a state of acute infection. However, lower expression of *S100A8* and *S100A9* in the H_L. reuteri+SL1344 group shows that L. reuteri SLZX19-12 can mitigate the infection and its induced inflammation. S100A8/S100A9 can induce the release of proinflammatory factors by activating the NF-κB signaling pathway (49). In line with results for S100A8 and S100A9, the upregulation of proinflammatory factors TNF-α and IL-1β and nuclear NF-κB P50 in mice of the SL1344 group and their downregulation in the H_L. reuteri+SL1344 group further confirm that the S100A8/S100A9–NF-κB pathway played an important role in infection of mice induced by *S.* Typhimurium SL1344. In addition, L. reuteri SLZX19-12 conferred a lower activation level of the S100A8/S100A9–NF-κB pathway and thus less severe inflammatory damage in mice. As an anti-inflammatory factor, decreased expression of the IL-10 gene in the SL1344 group also reflects disabled immune homeostasis and upregulated proinflammatory response, which was also consistent with the results of the activated S100A8/S100A9–NF-κB pathway. IL-10 can suppress the bactericidal response of immune cells against *S.* Typhimurium, and its reduction promotes a defensive reaction but leads to the development of colitis (50, 51).

Leakage through the intestinal barrier caused by *S.* Typhimurium SL1344 infection and inflammation is directly related to the death of epithelial cells, in which pyroptosis is involved (52, 53). However, our results indicate that potential apoptosis may also play a role in this process. Gene and protein expression levels of the antiapoptosis protein BCL-2 decreased and increased in the SL1344 group and H_L. reuteri+SL1344 group, respectively. Even though actual apoptosis was not shown in this research, these changes indirectly suggest that L. reuteri SLZX19-12 supplementation may reduce the potential apoptosis of colonic cells induced by infection with *S.* Typhimurium SL1344. At the same time, the contrasting changes in the proapoptotic protein Bax also indicate the potential antiapoptosis effect of L. reuteri SLZX19-12.

### L. reuteri SLZX19-12 enhances the stability of the colonic microbiota to inhibit infection with *S.* Typhimurium SL1344.

Researchers debate whether microbial balance acts to limit pathogen load and consequently protect gut barrier function. Based on the loads of Salmonella in colonic chyme, we inferred that the minor damage observed by supplementation of L. reuteri SLZX19-12 was mainly due to the inhibition of microbial dysbiosis and to colonization by and proliferation of *S.* Typhimurium SL1344. Therefore, we further analyzed the colonic microbiota of mice by 16S rRNA gene sequencing. As shown by the Sobs and Chao indexes, precolonization with L. reuteri SLZX19-12 significantly increased α diversity of the colonic microbiota. Elevated α diversity correlates with enhanced ability to resist disturbance (54). According to the β diversity evaluated by PCoA, the structure of the colonic microbiota of the SL1344 group was changed dramatically. Indeed, *Deferribacteres*, *Lachnospiraceae*, *Ruminococcaceae*, and *Alistipes* increased but *Bacteroidetes*, *Muribaculaceae*, and *Dubosiella* declined in the abundance of the SL1344 group.

*Deferribacteres* contribute to the development of intestinal inflammatory diseases. In inflammatory bowel disease of piglets and rats, abundance of *Deferribacteres* markedly increases (55, 56). *Alistipes* is a facultatively pathogenic bacterium and facilitates the development of inflammation, obesity, and bowel cancer (57–59). Therefore, *S.* Typhimurium SL1344 disturbed the microbial structure of colon and increased the proportion of harmful bacteria. At the same time, dysbiosis of the microbiota caused by *S.* Typhimurium SL1344 may contribute to the destruction of intestinal barrier function. Compared to the SL1344 group, the H_L. reuteri+SL1344 group showed a higher proportion of *Bacteroidetes*, *Muribaculaceae*, and *Dubosiella* and a lower proportion of *Deferribacteres*, *Lachnospiraceae*, and *Ruminococcaceae*, whose levels were similar to those in the Con group. According to the PCoA, the colonic microbiota structure in the H_L. reuteri+SL1344 group was similar to that in the Con group, which suggests that L. reuteri SLZX19-12 enhanced the stability of the colonic microbiota. Therefore, a lower level of *S.* Typhimurium SL344 and its blunted effect on microbiota disorder may be the main reasons for the healthier colon of the H_L. reuteri+SL1344 group.

*Alloprevotella* was abundant in the H_L. reuteri+SL1344 group but was present at very low levels in the Con and SL1344 groups. It can be inferred that the increased level of *Alloprevotella* induced by L. reuteri SLZX19-12 may be one of the reasons for stronger stability, but such causality was not proved, and this needs further research. Previous research also found that the presence of *Alloprevotella* was related to maintaining stability of the microbiota (60), which is consistent with our findings. In this process, the enhanced production of SCFAs by microbiota, propionate in particular, may play major roles. *Alloprevotella* participates in the degradation of dietary fiber by breaking it down into monosaccharides, and then dominant SCFA-producing bacteria ferment monosaccharides to SCFAs (61). Therefore, based on the correlation analysis, the *Alloprevotella* may provide precursors to *Dubosiella* and norank_f_*Muribaculaceae* to produce propionate to maintain the health of the colon. Indeed, some studies also found that norank_f_*Muribaculaceae* abundance was strongly correlated with propionate, and bacteria in the family *Muribaculaceae* may produce propionate as a fermentation end product (62, 63). Further research is needed, and there is no definitive result. Therefore, protecting the intestinal mucosa from damage is a key step in the process of disease prevention. The stability of the gut microbiota is essential for integrity of the intestinal barrier (64).

### Conclusion.

In summary, infection with *S.* Typhimurium SL344 disrupts colonic microbiota and breaks down the intestinal barrier, leading to intestinal inflammation, potential apoptosis, and systemic infection. Supplementation with L. reuteri SLZX19-12 protects the colon from infection by enhancing the stability of the gut microbiota and barrier integrity and reducing inflammation.

## MATERIALS AND METHODS

### Animals and bacterial strains.

Thirty-seven SPF male C57BL/6J mice (3 weeks old) with similar body weights of about 11.5 g were purchased from Beijing HFK Bioscience Co., Ltd. All the trials involving mice complied with the guidelines of and were approved by the Institutional Animal Care and Use Committee of China Agricultural University (AW72011202-1-1). L. reuteri SLZX19-12 was isolated from the feces of Tibetan piglets. In order to identify the protective effect of L. reuteri SLZX19-12, the pathogen *S.* Typhimurium SL1344 from Beina Biotechnology Co., Ltd. (Beijing, China), was used to challenge the mice.

### Isolation of L. reuteri SLZX19-12.

To isolate L. reuteri, 1 g feces was diluted in 10 mL phosphate-buffered saline (PBS) and homogenized. One milliliter of homogenized mixture was added to 50 mL MRS liquid medium with a pH of 4.0 and was cultured under anaerobic conditions for 24 h to enrich L. reuteri. The MRS liquid medium contained 10 g/L soluble starch, 10 g/L peptone, 4 g/L yeast extract powder, 5 g/L sodium acetate, 2 g/L KH_2_PO_4_, 2 g/L ammonium citrate, 0.2 g/L MgSO_4_·7H_2_O, 0.05 g/L MnSO_4_·7H_2_O, and 1 mL/L Tween 80. Subsequently, 50, 100, 150, and 200 μL cultured bacterial solution were spread evenly on MRS agar plates with added bromocresol blue by shaking with sterile glass beads. Monoclonal bacterial colonies of acid-producing bacteria with different morphologies were selected for purification by scribing and anaerobic culture on plates. Monoclonal bacterial colonies were selected for culture, and bacterial DNA was extracted for PCR to acquire full-length 16S rRNA. The PCR products (about 1,500 bp) were sequenced with a 3730xl DNA analyzer. The identities of the bacterial strains were confirmed by conducting a BLAST search with the nucleotide collection (nr/nt) of the NCBI database and multiple biochemical tests.

### Physiological and biochemical tests of L. reuteri SLZX19-12.

The growth curve of L. reuteri SLZX19-12 was plotted by reading the optical density at 600 nm (OD_600_) at different time points, for which the number of bacteria was obtained by counting colonies on MRS agar plates. The acid tolerance and bile salt tolerance tests were performed by adding hydrochloric acid and bile salt, respectively, to the desired pH or concentrations. A biochemical detection kit for lactic acid bacteria (Hopebio; SHBG13) and an API-ZYM enzymatic detection kit (bioMérieux; API-ZYM) were used to evaluate characteristics of L. reuteri SLZX19-12. All procedures followed the instructions provided by kit manufacturers. Antibiotic resistance was tested with antibiotic-sensitive paper (Hangzhou Microbial Reagent Co., Ltd.; S1051-S1074). After the L. reuteri SLZX19-12 solution was spread evenly on MRS agar plates with a sterile spreading rod, the antibiotic susceptibility papers were put on the predetermined area. After 24 h of growth, the presence of inhibition zones was noted and their diameters were measured.

### Treatment of mice and sample collection.

Monoclonal L. reuteri SLZX19-12 was inoculated into MRS medium (20 mL) and anaerobically cultured for 12 h. The cultured bacterial solution was centrifuged at 100 × *g* for 10 min and resuspended with normal saline. The solution was successively diluted to 1 × 10^7^ CFU/mL (low dose, for the L-L. reuteri group), 1 × 10^8^ CFU/mL (medium dose, for the M-L. reuteri group), and 1 × 10^9^ CFU/mL (high dose, for the H-L. reuteri group) with normal saline. Each group contained 4 mice. Fifty microliters of sodium bicarbonate (0.05 g/mL) was administered to neutralize gastric acid, and then 150 μL normal saline (Con group) or L. reuteri SLZX19-12 solution was immediately injected into the stomachs of the mice with gastric injection needles. The gavage was given once every 2 days for 14 days until sample collection. To determine the effects of L. reuteri on *S.* Typhimurium SL1344 infection, 150 μL normal saline (for the Con and SL1344 groups) or L. reuteri SLZX19-12 with 1 × 10^9^ CFU/mL (for the H_L. reuteri+SL1344 group) was administered by gavage to mice to establish colonization. Similarly, the gavage was given once every 2 days for 14 days. Then, 150 μL normal saline (for the Con group) or *S.* Typhimurium SL1344 solution with 1 × 10^6^ CFU/mL (for the SL1344 and H_L. reuteri+SL1344 groups) was administered by gavage to the mice. Each group contained 7 mice. After 1 week, samples were collected. During the experiment, mice were housed in an environmentally controlled room (24 ± 1°C, 50 to 60% relative humidity, 12-h light-dark cycle). Samples collected included serum, feces, chyme, ileum, colon, spleen, and liver. Blood was collected from the eyeball and then centrifuged at 400 × *g* to acquire the serum. Samples other than serum were collected in Eppendorf tubes, rapidly frozen in liquid nitrogen, and then held at −80°C. Chyme, liver, and spleen were used to determine pathogen loads and were temporarily stored on ice, and detection was carried out as soon as possible. Intestinal tissues for morphological examination were fixed in 4% paraformaldehyde. The relative weights of liver and spleen were calculated as liver weight/body weight and spleen weight/body weight, respectively. The length of the colon was measured with a ruler.

### Disease activity index score.

Parameters associated with infection, such as weight loss and rectal bleeding, were used to calculate the disease activity index (DAI). Each mouse was evaluated and assigned a DAI based on the average values according to the guidelines presented in Table S1.

### Histological score.

The tissues from the fixed middle position of the colon were acquired for sectioning and HE staining. The levels of inflammatory cell infiltration, pathological damage to crypt structure, ulceration of epithelium, and edema were evaluated to acquire the histological score. The histological scores in detail are shown in Table S2.

### Detection of Salmonella loads in colonic chyme and visceral organs.

Colonic chyme, livers, and spleens were added to sterile normal saline at ratios of 0.02 g/mL, 0.1 g/mL, and 0.04 g/mL, respectively. After homogenizing with 1-mm sterile steel balls, each mixture was serially diluted to 1×, 10×, 100×, 10^3^×, 10^4^×, and 10^5^×. One hundred microliters of each dilution was spread evenly on Salmonella-*Shigella* (SS) agar plates with sterile glass beads. SS agar medium contained 5 g/L beef powder, 5 g/L tryptose, 10 g/L lactose, 8.5 g/L bile salt no. 3, 8.5 g/L sodium citrate, 8.5 g/L sodium thiosulfate, 1 g/L ferric citrate, 0.025 g/L neutral red, 0.33 mg/L brilliant green, and 17.0 g/L agar. After aerobic culture for about 18 h at 37°C, black positive colonies were counted. Viable colony counts ranged from 30 to 300 per plate. Accordingly, loads of Salmonella in the colonic chyme, livers, and spleens were calculated and analyzed.

### Detection of LPS and IL-1β content by ELISA.

The LPS and IL-1β concentrations in serum and colons were detected by a commercial mouse LPS enzyme-linked immunosorbent assay (ELISA) kit (Cusabio; CSB-E13066m) and a mouse IL-1β ELISA kit (Cusabio; CSB-E08054m). All the procedures followed instructions provided by the manufacturers of the ELISA kits.

### 16S rRNA gene sequencing and analysis.

Colon microbial genomes were extracted by a commercial E.Z.N.A. stool DNA kit (Omega; D4015-01), and quality was verified by 1% agarose gel electrophoresis. The microbial V3+V4 region of the 16S rRNA gene was amplified by universal primers (338F [5′-ACTCCTACGGGAGGCAGCAG-3′] and 806R [5′-GGACTACHVGGGTWTCTAAT-3′]) with barcoding to produce amplified fragments of about 500 bp. PCRs were conducted with FastPfu DNA polymerase (TransGen; AP221) and an ABI GeneAmp 9700 PCR amplifier for 30 cycles of predenaturation at 95°C for 3 min, denaturation at 95°C for 20 s, annealing at 58°C for 15 s, and extension at 72°C for 20 s, followed by 1 cycle at 72°C for 5 min. PCR amplification products were detected by 2% agarose gel electrophoresis and then recovered using an AxyPrep DNA gel extraction kit (Axygen Biosciences, USA). After product quantification and homogenization, DNA was denatured with sodium hydroxide to produce single-stranded DNA. The constructed MiSeq library was sequenced using the Illumina PE300 platform. The paired-end reads obtained were merged by Flash software (version 1.2.11), and the sequence data were quality controlled and filtered. Clustering of operational taxonomic units (OTU) was performed on the optimized sequence by UPARSE (version 7.1) based on 97% similarity. The representative sequences of OTU were annotated using the Silver database with RDP Classifier (version 2.2; confidence threshold value, 0.7).

### Detection of SCFAs by ion chromatography.

Short-chain fatty acid (SCFA) concentrations in feces were quantified by an ion chromatography method. Briefly, about 25 mg feces from mice was added to 4 mL ultrapure water. After homogenization with clean glass beads, samples were placed in an ultrasonic bath (30 min) and then centrifuged at 10,000 × *g* at 4°C for 10 min. The supernatant was directly filtered with 0.22-μm mesh. All filtrate was analyzed by a Dionex ICS-3000 ion chromatography system (Dionex, Sunnyvale, CA, USA) with a Bio-Rad Aminex HPX-87H column. The condition of 1.8 mM heptafluorobutyric acid (HFBA) at a column temperature of 42°C was used for elution with a flow rate of 0.6 mL/min. Analysis time was 20 min. Formate, lactate, acetate, propionate, butyrate, isobutyrate, valerate, and isovalerate were all quantified using standard curves based on the appropriate standards.

### Gene expression by real-time quantitative PCR.

RNA of colon tissues was extracted using a commercial kit (CWBiotech; CW0584S), and RNA concentration and quality were evaluated with an ultra-micro spectrophotometer (NanoDrop 2000; Thermo Fisher) and 1% agarose gel electrophoresis. One thousand nanograms of RNA was reverse transcribed to cDNAs using a HiFiScript reverse transcriptase mixture with genomic DNA remover (CWBiotech; CW2020M). The SYBR green (CWBiotech; CW0659) method was used to quantify gene expression levels with a real-time quantitative PCR (RT-qPCR). The 10-μL mixture including 0.2 μM primer and 2 μL diluted cDNAs was run on the LightCycler 96 RT-qPCR system (Roche), followed by predenaturation at 95°C for 60 s and 40 cycles of denaturation at 95°C for 15 s, annealing at 60°C for 15 s, and extension at 72°C for 45 s. GAPDH was used as an internal reference. All the primers for target genes are listed in Table S3. Standard and dissolution curves were obtained to confirm efficiency and specificity of the primers. The 2^−ΔΔ^*^CT^* method was used to calculate relative expression level of target genes.

### Protein expression by Western blotting.

About 50 mg colonic tissue was placed in radioimmunoprecipitation assay (RIPA) lysis buffer (Beyotime; P0013B) containing phenylmethylsulfonyl fluoride (PMSF) and phosphatase inhibitors and was homogenized with a homogenizer at −20°C. After centrifugation at 12,000 × *g* for 10 min, protein concentrations of the supernatant were measured using a bicinchoninic acid (BCA) assay kit (Thermo Fisher; A53225). Nuclear proteins from colon tissues were extracted using a commercial nuclear and cytoplasmic protein extraction kit (Beyotime; P0027). All samples were diluted to 3 μg/μL solution with RIPA lysis buffer and SDS-PAGE loading buffer (Beyotime; P0015). After protein denaturation at 100°C for 10 min, samples were subjected to SDS-PAGE electrophoresis, in which about 60 μg proteins was added. The electrophoretic buffer consisted of 14.4 g/L glycine, 3.03 g/L Tris, and 1 g/L SDS in deionized water. Conditions for electrophoresis and transfer to the membrane were constant at 20 mA and 200 mA, respectively. Transfer membrane buffer included 14.4 g/L glycine, 3.03 g/L Tris, and 20% methanol (vol/vol) in deionized water.

After the proteins were transferred to 0.2 μm PVDF membranes, these membranes were blocked with 5% skim milk powder in PBS for 2 h at room temperature. Then the membranes were incubated overnight in primary antibodies diluted 1:1,000 with 5% skim milk powder solution. Antibody solution contained primary antibodies for S100A9 (Beyotime; AF7932), TNF-α (Abcam; ab183218), NF-κB P50 (CST; 13586S), claudin-3 (Abcam; ab15102), claudin-7 (Abcam; ab27487), occludin (Abcam; ab31721), Bcl-2 (Proteintech; 26593-1-AP), Bax (Proteintech; 60267-1-Ig), GAPDH (SAB; 21612), β-tubulin (Beyotime; AF1216), and PCNA (Santa Cruz; SC-56). After three washes for 5 min each, fluorescently labeled secondary antibodies, including donkey anti-mouse IgG (LI-COR; 926-32212) or goat anti-rabbit IgG (CST; 5151S), were incubated with the membranes for 4 h at 4°C. After three washings, the membranes were read by an Odyssey infrared imager (LI-COR, USA). Tubulin and GAPDH were used as internal references to correct loading errors for total proteins, while PCNA was used as an internal reference for nuclear proteins.

### HE staining of colon sections.

Colonic tissues fixed in 4% paraformaldehyde were removed and neatly trimmed. Segments (0.5 cm) of tissues were placed in embedding boxes and washed overnight with running water. The tissues in the embedding boxes were dehydrated with an increasing gradient of ethanol from 70% to 100%. After treatment with xylene, colonic tissues were immersed in paraffin at 65°C for 2 h and then embedded by cooling. Subsequently, tissues were cut into 0.2-μm slices, and slices were fixed to antislip slides. These samples were dewaxed with xylene and treated with a reducing gradient of ethanol ranging from 100% to 70%. To complete the rehydration process, samples were rinsed with tap water for 2 min. Next, the samples were stained with hematoxylin solution (5 g/L) for 5 min and immersed in 1% hydrochloric acid for 10 s to separate the staining. After a second staining with eosin aqueous solution for about 15 s, the samples were rapidly dehydrated with a gradient ethanol aqueous solution from 70% to 100% and treated in xylene for 10 min to finish the lucency process. Finally, slices were sealed with cedar oil and observed with the BX 53 microscope system (Olympus, Japan).

### Statistical analysis.

All data are presented as means and standard errors (SE). Contrast analysis was conducted to analyze differences. In the 16S rDNA sequencing analysis, the α diversity was evaluated by Chao, Sobs, Shannon, and Simpson indexes. The β diversity was analyzed by PCoA based on the weighted normalized UniFrac method. Diversity analysis and data visualization were performed using Qiime2 software. Correlation analysis was based on Spearman’s rank correlation coefficient and was carried out by R (version 3.3.1, pheatmap package). Other statistics and plots were constructed using SPSS 24 (SPSS Inc., Chicago, IL, USA) and GraphPad Prism 8.0.1 software (GraphPad Software Inc., USA). *P* values ranging from 0.05 to 0.10 are considered to indicate a tendency.

### Data availability.

Sequencing data generated in this study were deposited in Sequence Read Archive (SRA) with the SRA accession numbers SRR15092578 to SRR15092596. The raw data supporting the conclusions of this article will be made available by the authors without undue reservation.
