# Supplementary material for: Limosilactobacillus reuteri SLZX19-12 Protects the Colon from Infection by Enhancing Stability of the Gut Microbiota and Barrier Integrity and Reducing Inflammation
Source: Microbiol Spectr. 2022 Jun 6;10(3):e02124-21. doi: 10.1128/spectrum.02124-21 (PMC9241593; doi:10.1128/spectrum.02124-21)
Supplement: Supplemental file 1 — Tables S1 to S3; Fig. S1 and S2. Download spectrum.02124-21-s0001.pdf, PDF file, 0.6 MB [file spectrum.02124-21-s0001.pdf]

Table S1. Scoring standard for calculating a disease activity index based on weight loss and the degree of intestinal bleeding.

Score	Weight loss	Bloody stool score
0	None	None
1	1–5%	None
2	6–10%	Positive hemocult
3	11–15%	Visible blood traces in stool
4	16–20%	Obvious blood traces in stool
5	>20%	Gross rectal bleeding

Table S2. Scoring standard for colon histology.

Score	Inflammation	Crypt structure	Ulceration	Edema
0	No significante infiltration	None	None	None
1	A few inflammatory cells in mucosa	A few crypt damaged and distorted	Deformed or folded epithelium	Present but less
2	A few inflammatory cells in mucosa and submucosa	Some spaces between crypts, loss of goblet cells	Damaged epithelium	Marked cellular swelling
3	Obvious infiltration in mucosa and submucosa	Large spaces between crypts and loss of 1/3 goblet cells	Small ulceration	Many cellular swelling
4	Large amount of infiltrations in most area of mucosa and submucosa	Large spaces between crypts and loss of 1/2 goblet cells	Frequent small ulceration	Most cellular swelling
5	Transmural inflammation from mucosa to muscle layer	No significant crypts or loss of most of the goblet cells	Large areas of ulceration	Most dilated and rounded cells

Table S3. Specific primers of related genes[1].

Gene	Accession No.	Sequences (5'→3')	Product size (bp)
Bcl-2	NM_001355053.1	Forward: TCATTTCCCATCCCGCTGT<br>Reverse: GGTCCTTGTCTCATTATCCCA	134
EpCAM	NM_008532.2	Forward: GTCATTGTGGTGGTGTCATTAG<br>Reverse: CATCTCCTTTATCTCAGCCTTC	105
IL-1β	NM_008361.4	Forward: ACCTGTGTCTTTCCCGTGG<br>Reverse: TCATCTCGGAGCCTGTAGTG	162
IL-6	NM_001314054.1	Forward: GAGCCCACCAAGAACGATA<br>Reverse: TTGTCACCAGCATCAGTCC	102
IL-10	NM_010548.2	Forward: TGGACAACATACTGCTAACCG<br>Reverse: GGGCATCACTTCTACCAGGT	107
IL-17a	NM_010552.3	Forward: GCTCCAGAAGGCCCTCAGA<br>Reverse: CTTTCCCTCCGCATTGACA	140
IL-18	NM_008360.2	Forward: ACAACTTTGGCCGACTTCAC<br>Reverse: GGGTTCACTGGCACTTTGAT	128
IL-22	NM_016971.2	Forward: TCCGAGGAGTCAGTGCTAAA<br>Reverse: AGAACGTCTTCCAGGGTGAA	73
IL-23a	NM_031252.2	Forward: TGTGCCCCGTATCCAGTGT<br>Reverse: CGGATCCTTTGCAAGCAGAA	81
IFN-γ	NM_008337.4	Forward: GCTCTGAGACAATGAACGCTACAC<br>Reverse: TTCTTCCACATCTATGCCACTTGAG	149
GAPDH	NM_001289726.1	Forward: TGTTCCTACCCCCAATGTGT<br>Reverse: GGTCCTCAGTGTAGCCCAAG	137
LC3-α	NM_025735.3	Forward: AACATGAGCGAGTTGGTCAAGAT<br>Reverse: GTCTTCATCCTTCTCCTGTTCATAG	144
REGIIIγ	NM_011260.2	Forward: ATGCTTCCCCGTATAACCATCA<br>Reverse: GGCCATATCTGCATCATACCAG	201
S100A8	NM_013650.2	Forward: ATCCTTTGTCAGCTCCGTCTTC<br>Reverse: GGGCATGGTGATTTCCTTGTATATT	140
S100A9	NM_009114.3	Forward: GAAGGAAGGACACCCTGACAC<br>Reverse: TTTATGAGGGCTTCATTTCTCTTCTC	108
TGF-β	NM_011577.2	Forward: ACCGCAACAACGCCATCTA<br>Reverse: TGCTTCCCGAATGTCTGACG	86
TNF-α	NM_013693.3	Forward: CCACGCTCTTCTGTCTACTG<br>Reverse: ACTTGGTGGTTTGCTACGA	169

[1] TNF-α: tumor necrosis factor-α; IL-1β: interleukin-1β; EpCAM: epithelial cell adhesion molecule; LC3, light chain 3; INF-γ: interferon-γ; TGF-β: transforming growth factor-β; REGIIIγ: regenerating islet-derived protein IIIγ; Bcl-2: B-cell lymphoma-2.

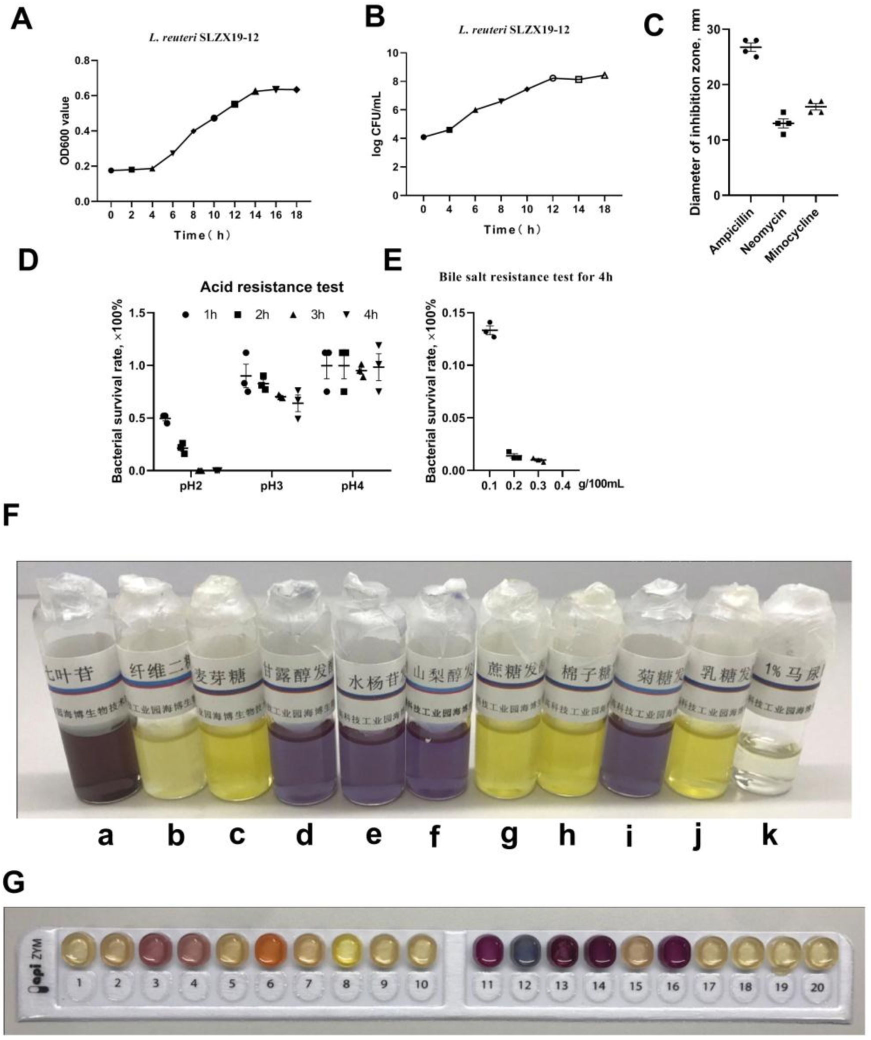

FIG S1 The detection of physiological and biochemical characteristics of *L. reuteri* SLZX19-12. (A) Growth curve of *L. reuteri* SLZX19-12 based on OD_{600} values. (B) The numbers of *L. reuteri* SLZX19-12 at different time. (C) Diameters of inhibition zones under the effects of fluorine ampicillin, neomycin and minocycline susceptibility papers. (D) The survival rates of *L. reuteri* SLZX19-12 in different pH environments. (E) The survival rates of *L. reuteri* SLZX19-12 in different concentrations of bile salt. (F) The results of biochemical identification of *L. reuteri* SLZX19-12. This detection was finished with a biochemical detection kit for lactic acid bacteria. a: aesculin; b: cellobiose; c: maltose; d: mannitol; e: salicin; f: sorbitol; g: sucrose; h: raffinose; i:inulin; j: lactose; k: sodium equurate. The yellow and purple show positive and negative, respectively. (G) Results of enzyme spectrum analysis of the *L. reuteri* SLZX19-12. This detection was finished with an API-ZYM enzymatic detection kit. 1: Control; 2: Alkaline phosphatase; 3: Esterase C4; 4: Lipase like C8; 5: Lipase like 14; 6: Albumine arylaminase; 7: Valine arylaminase; 8: Cystine arylaminase; 9: Trypsin; 10: Chymotrypsin; 11: Acid phospholipase; 12: Naphthol AS-BI-phosphate hydrolase; 13: α-galactosidase; 14: β-galactosidase; 15: β-glucuronidase; 16: α-glucosidase; 17: β-glucosidase; 18: N-acetyl-glucosaminase;

19: α-mannosidase; 20: β-alginosidase. The Orange shows negative as a control, and the blue or blue-purple shows positive.

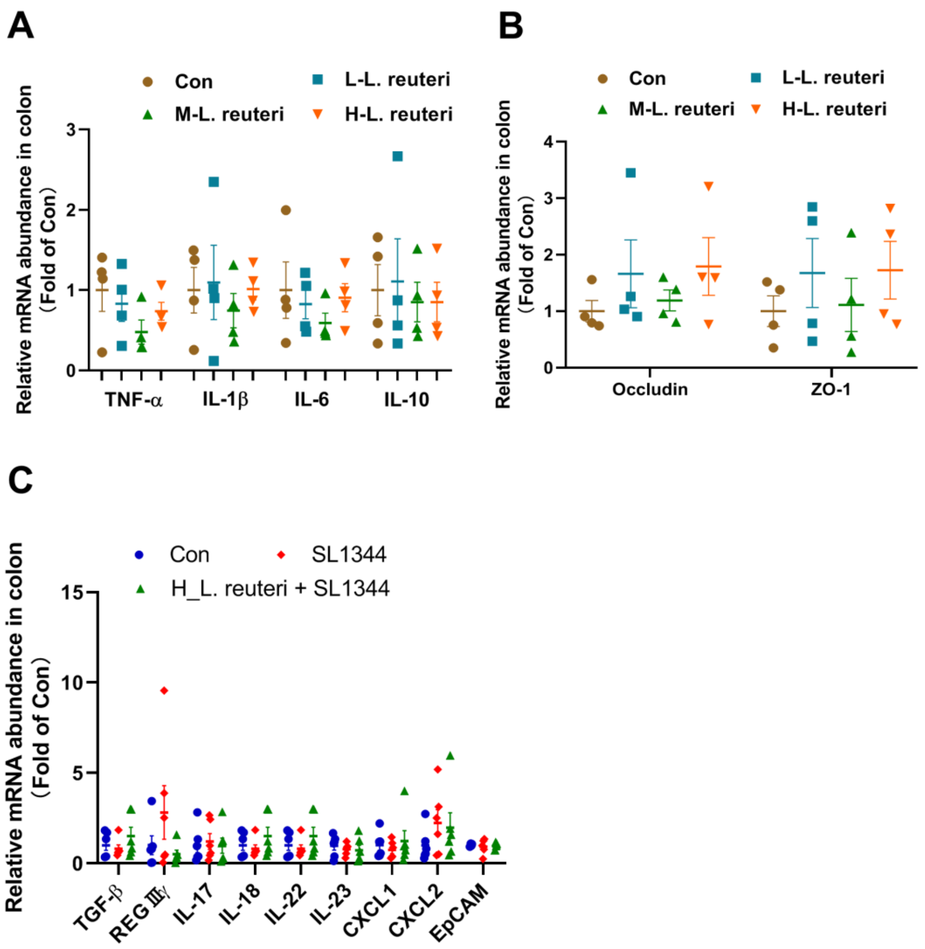

FIG S2 The genes expression levels of colonic immune and barrier related proteins of mice treated with individual *L. reuteri* SLZX19-12 or in anti-infection model induced by *S. typhimurium* SL1344. (A) The relative genes expression levels of pro-inflammatory factors in colon of mice treated with *L. reuteri* SLZX19-12. These mice were treated by vehicle (Con group) or a low dose (L-L. reuteri group), medium dose (M-L. reuteri group), or high dose (H-L. reuteri group) of *L. reuteri* SLZX19-12, respectively. Mean ± SE are shown (n = 4). (B) The relative genes expression levels of barrier protein *Occludin* and *ZO-1* in colon of mice. Mean ± SE are shown (n = 4). (C) The relative genes expression levels of colonic immune and barrier related proteins of mice. These mice were treated by vehicle (Con and SL1344 group) or a high dose of *L. reuteri* SLZX19-12 (H_L. reuteri group + SL1344 group), and then infected with vehicle (Con group) or *S. typhimurium* SL1344 (SL1344 and H_L. reuteri group + SL1344 group). Mean ± SE are shown (n = 6).
